# A Case of Hypomagnesemia Presenting as New-Onset Seizure

**DOI:** 10.7759/cureus.23791

**Published:** 2022-04-03

**Authors:** George Michael, Kitty George

**Affiliations:** 1 Orthopedic Surgery, Thrissur Government Medical College, Mulakunnathukavu, IND; 2 Internal Medicine, Patient First Medical Center, Dearborn, USA; 3 Internal Medicine, MES Medical College, Perinthalmanna, IND; 4 Obstetrics and Gynecology, Memorial Women's Specialists, Houston, USA

**Keywords:** vitamin-d deficiency, thiazide diuretics, proton pump inhibitor, hypocalcemia, hypokalemia, electrolyte abnormalities, diarrhea, renal failure, hypomagnesemia, seizure

## Abstract

Identifying the etiology of a new-onset seizure can be challenging due to the extraordinarily large number of differential diagnoses. Electrolyte disturbances, through a mechanism of disruption of neurotransmission within the central nervous system, are often implicated as a possible etiology in many cases of seizures. In this report, we describe the sequence of events related to a 68-year-old patient who suffered from convulsions precipitated by hypomagnesemia and the possible contributors to such a condition.

## Introduction

Magnesium, a divalent cation, is one of the major intracellular electrolytes [1]. It is a cofactor for various intracellular enzymes and plays a significant role in energy production by forming complexes with adenosine triphosphate (ATP) molecules [2]. Along with calcium, it is also linked to neuromuscular transmission, which takes place extracellularly. It is interesting to note that the level of magnesium in the serum, which we can measure in the normal range of 1.7-2.4 mg/dL [3], accounts for only 0.3% of the total body magnesium, making it a poor predictor of intracellular magnesium content [4].

## Case presentation

Our patient, a 68-year-old female of Middle Eastern ethnicity with no history of any prior episodes of convulsions, was brought to the emergency department after an observed seizure event while sleeping at night. On arrival, she was in a typical post-ictal state in the form of confusion and drowsiness. Her recent past medical history revealed that she had been treated conservatively for suspected viral gastroenteritis two days prior when she had presented with nausea, vomiting, abdominal discomfort, and diarrhea. All her symptoms except for diarrhea had subsided by the time she sustained a seizure episode. Consequently, the patient had been admitted, and routine investigations had been performed. During her hospital stay, she had experienced two more episodes of observed seizures, which had been identified as the generalized tonic-clonic type, each lasting for less than two minutes. Her other medical conditions included pre-diabetes, hypertension, dyslipidemia, osteoarthritis, gastroesophageal reflux disease (GERD), and asthma, for which she was treated with dietary modifications, losartan-hydrochlorothiazide, atorvastatin, hydrocodone-acetaminophen, omeprazole, and albuterol, respectively. She also used to take over-the-counter vitamin supplements that contained zinc.

Upon admission to the hospital, the patient was afebrile and had near-normal vital signs. Her blood investigations revealed severe hypomagnesemia (0.6 mg/dL) and deranged kidney function with serum creatinine elevation to 3.02 mg/dL, significantly above her baseline of less than 1 mg/dL. Her serum potassium and calcium levels were 3.2 mg/dL and 6.6 mg/dL, respectively. Urinalysis showed epithelial cell casts, urine sodium of 103 mEq/L, and urine potassium of 12.4 mEq/L. MRI brain showed no acute intracranial pathologies, and EEG was found to be normal. A 12-lead ECG was also taken, which showed no abnormalities except for the corrected QT interval of 461 ms. A summary of the patient's various lab findings is shown in Table 1.

**Table 1:** Lab results

Component	Reference range	2/7/2022	2/9/2022	2/21/2022
Sodium	135–145 mmol/L	138	140	142
Potassium	3.5–5.2 mmol/L	3.2	3.3	4.7
Chloride	98–111 mmol/L	102	104	105
Anion gap	5–17 mEq/L	18	15	14
Bicarbonate	21–29 mmol/L	21	24	28
Glucose	60–99 mg/dL	121	138	105
Blood urea nitrogen (BUN)	7–25 mg/dL	19	32	25
Creatinine	0.50–1.10 mg/dL	3.02	2.85	1.22
Glomerular filtration rate (GFR)	Non-African American: ≥60 mL/min/1.73 m^2^	22	44	58
Calcium	8.5–10.5 mg/dL	6.6	8.4	9.6
Magnesium	1.7–2.4 mg/dL	0.6	1.4	1.7
Phosphorus	2.5–4.5 mg/dL	4.6	3.8	4
Protein total	6.2–8.1 g/dL	7.2	7	7.1
Albumin	3.5–4.9 g/dL	3.9	4	4.3
Globulin	2.2–3.7 g/dL	3.3	3.1	2.8
Alkaline phosphatase	37–135 U/L	88	79	49
Aspartate aminotransferase (AST)	<35 U/L	27	30	27
Alanine aminotransferase (ALT)	8–37 U/L	23	32	44
Bilirubin total	0.3–1.2 mg/dL	0.6	0.6	0.4
Vitamin D (25-Hydroxy)	20–50 ng/mL	-	16	-

Treatment with lorazepam controlled the seizures, and levetiracetam was added for seizure prophylaxis after neurology consultation. The patient was started on intravenous fluids, magnesium, and calcium. The very next day, her diarrhea subsided, and over the next two weeks, her magnesium levels returned to normal range, and her calcium and potassium reverted to normal, along with her renal function. Her blood pressure medications were changed to amlodipine, and omeprazole was stopped. The patient was given vitamin D supplementation as well.

## Discussion

The causes of hypomagnesemia can be broadly classified into four main categories: impaired intestinal absorption, increased intestinal loss, increased renal loss, and rapid shifts from the extracellular fluid [5]. Dietary magnesium deficiency is not a common condition except in alcohol-dependent patients who are also severely malnourished. Our patient had never used alcohol but had vomiting and diarrhea, which can lead to excessive loss of magnesium from the intestine. The volume-depleting effect of severe diarrhea could have also contributed to the failure of renal reabsorption of magnesium by the precipitation of acute renal failure. However, what made this case more striking was the combined effect of the various medications the patient was taking for her chronic conditions. The proton pump inhibitor (PPI), which she had been taking for GERD for more than 10 years, has been implicated in several studies as a causative agent of hypomagnesemia by a non-renal mechanism. Loop and thiazide diuretics can decrease renal magnesium reabsorption [6] via the paracellular pathway by abolishing the net luminal positive charge created by sodium chloride reabsorption in the renal tubules. Vitamin supplements containing zinc are also known to reduce magnesium levels. The development of metabolic acidosis can further aggravate hypomagnesemia by driving the magnesium intracellularly. Unfortunately, arterial blood gas monitoring was not done in the patient during the acute stage, resulting in an inability to measure the actual pH level. We believe the patient might have had a chronic state of magnesium depletion due to the effects of medications like PPI and diuretics, which got aggravated by acute loss and acute kidney injury following viral gastroenteritis. All these factors contributed in unison to severe hypomagnesemia in the patient.

**Figure 1:**
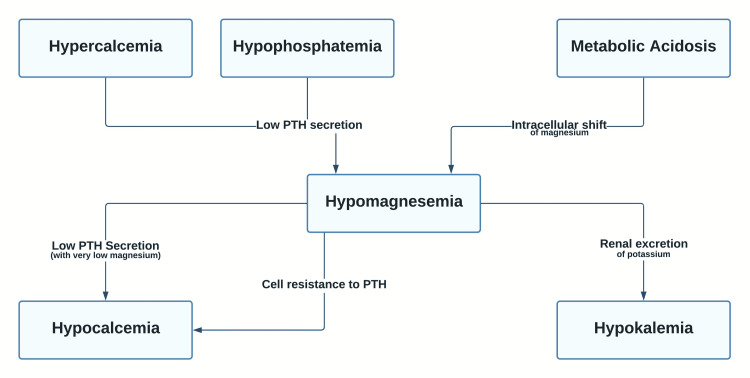
Hypomagnesemia and other electrolyte abnormalities PTH: parathyroid hormone

Hypomagnesemia is often asymptomatic until the levels are less than 1.2 mg/dL. A decrease in the extracellular magnesium causes a greater influx of calcium, resulting in an even greater release of neurotransmitters. Increased glutamate-mediated depolarization in the brain can cause seizures. Hypomagnesemia can also lead to muscle cramps, weakness, tetany, and hyperreflexia. Apathy, irritability, delirium, depression, and psychosis are some of the neuropsychiatric manifestations of magnesium deficiency [7]. Cardiac arrhythmias are often preceded by ECG changes, including prolonged PR and QT intervals and T-wave flattening/inversion. Other potential manifestations include respiratory depression, urinary retention, and paralytic ileus. The features of hypomagnesemia also involve the characteristics of other associated electrolyte abnormalities, which are intricately linked to magnesium levels. Consequent to a severe deficiency of magnesium, increased renal potassium excretion occurs. This effect is primarily mediated by the renal outer medullary potassium (ROMK) channels, which are luminal channels. Because there is a high concentration of potassium intracellularly, a free leak of potassium occurs into the urine. Magnesium deficiency can also lead to inhibition of parathyroid hormone (PTH) secretion and increased cellular resistance to PTH, contributing to hypocalcemia [8]. A simplified version of the concept demonstrating the relationship between magnesium and other electrolytes is illustrated in Figure 1.

The assessment of magnesium status is usually done with the measurement of serum magnesium concentration. As mentioned before, the serum magnesium level is a poor predictor of the total body magnesium status. Magnesium levels are higher in the red blood cells, and hence even a slight amount of hemolysis can affect the estimation of serum magnesium. Of note, 24-hour excretion of magnesium in urine is a valuable tool in the assessment when renal wasting is suspected. The magnesium retention test is another niche method that measures the total body magnesium content based on the amount of magnesium retained by a loading test, which is increased in hypomagnesemic states.

Severe hypomagnesemia should be treated with intravenous magnesium salts with adequate dose reduction for renal insufficiency. It is increasingly being recommended to use the less toxic MgCl_2_ salts instead of MgSO_4_ [9] because the sulfate anions can bind calcium in the extracellular fluid and urine. It is equally important to consider vitamin D deficiency and other electrolyte abnormalities, which often occur together when treating magnesium deficiency. These concurrent abnormalities are difficult to treat until magnesium is repleted. Calcium supplementation during magnesium repletion therapy can help avoid the inadvertent activation of the PTH, leading to a rapid onset of hypophosphatemia and fatal complications like rhabdomyolysis. Vitamin D deficiency can critically limit magnesium absorption, and hence treatment should be initiated early. When supplementing vitamin D, it is often advisable to use the 25-(OH) vitamin D instead of the usual 1, 25-(OH)_2_ vitamin D (calcitriol) form because of the potential suppression of magnesium reabsorption by the renal tubules [10], which is mediated by the PTH.

## Conclusions

Hypomagnesemia is often underestimated as a potential cause of seizures, especially in the setting of multiple electrolyte abnormalities. Understanding the complex interrelationships between magnesium and other electrolytes is crucial in providing the appropriate therapy and preventing possible complications. Severe hypomagnesemia is often a medical emergency and, as such, should be recognized early, especially given the drastic implications it can have on the nervous and cardiorespiratory systems.
